# Unlocking the black box: multimodal imaging and quantitative analysis of plant vesicular trafficking

**DOI:** 10.1007/s44307-026-00101-2

**Published:** 2026-03-10

**Authors:** Yanyan Zhang, Changwen Xu, Xinxiu Zuo, Hongping Qian, Xi Zhang, Jinxing Lin, Yaning Cui

**Affiliations:** 1 State Key Laboratory of Tree Genetics and Breeding, College of Biological Sciences and Technology, Beijing Forestry University, Beijing, 100083 China; 2 National Engineering Research Center of Tree Breeding and Ecological Restoration, Beijing Forestry University, Beijing, 100083 China; 3 The Tree and Ornamental Plant Breeding and Biotechnology Laboratory of National Forestry and Grassland Administration, Beijing Forestry University, Beijing, 100083 China

**Keywords:** Vesicular trafficking, Live-cell imaging, Labeling techniques, Dynamics, Quantitative analysis

## Abstract

How do plants, lacking a central nervous system, translate environmental stimuli into physiological actions within milliseconds? Vesicular trafficking acts as a cellular core signal and material transport hub that facilitates this rapid adaptation, yet its dynamic nature has long remained a "black box". Traditional imaging approaches have struggled not only with optical resolution (the "unseen"), but critically with a lack of quantitative precision (the "immeasurable") and the inability to track molecular history (the "unknown age"). This review synthesizes a new paradigm that unlocks this black box by integrating advanced chemical biology with deep learning computational analysis. We detail how multimodal strategies combining pH-sensitive probes (e.g., pHluorin), covalent tags (HaloTag), and fluorescent timers visualize molecular events with unprecedented fidelity. Furthermore, we explore how integrating next generation FRAP/FCS variants (DeepFRAP, FCSNet) with deep learning allows for the rigorous mathematical modeling of vesicle kinetics. By resolving long-standing controversies such as endocytic stoichiometry and secretory sorting logic, this quantitative framework maps nanoscale membrane dynamics to organismal phenotypes, ultimately refining our understanding of plant stress resilience and signal transduction.

## Introduction

In the sessile life of plants, vesicular trafficking acts as a central signal transduction and material transport hub that converts environmental stimuli into physiological responses. In *Arabidopsis thaliana* in response to salt stress, the fusion between multivesicular bodies (MVBs) and vacuoles is impaired, while vacuolar morphological remodeling elevates the efficiency of Na^+^ compartmentalization. This process achieves cytosolic Na^+^ detoxification and thereby enhances plant salt tolerance (Liu et al. 2025a). In addition, the dynamic delivery of "stress cargos" via the transport of extracellular vesicles (EVs) derived from exocyst-positive organelle (EXPO) directly determines the capacity of plants to cope with pathogenic pathogens and salt stress (Gao et al. 2026). Far beyond a simple logistical system for material transport, the dynamic orchestration of endocytosis and exocytosis underpins the plant's ability to perceive pathogens, acquire nutrients, and reshape its architecture during development (Ling et al. 2025a; Madrid-Espinoza et al. 2025). For instance, the rapid recycling of FLS2 receptors is not merely a cellular housekeeping event but a critical checkpoint for pathogen detection (Cui et al. 2024), while the unconventional protein secretion (UPS) of RNAi-containing extracellular vesicles represents a sophisticated cross-kingdom weapon to silence fungal genes (Wang et al. 2025b).

However, a fundamental paradox has long plagued plant cell biology: while we have successfully cataloged the molecular players from clathrin coats to SNARE complexes, the spatiotemporal dynamics of these events remain largely a "black box". This opacity stems from the unique physical constraints of plant tissues. Unlike animal models, plant cells are encased in thick, refractive cell walls and packed with autofluorescent organelles like chloroplasts, which severely limit optical penetration and clarity (Chen et al. 2024b). Consequently, traditional imaging methods have struggled to capture the transient, nanoscale events of vesicle formation and fusion (Cui et al. 2019; Zhang et al. 2021), often resigning researchers to static snapshots or ensemble averages that obscure true molecular kinetics. Furthermore, the technical limitations of restrictive probes, such as phototoxicity and interference-prone fluorescence, have created bottlenecks for accurate, long-term monitoring in vivo (Hong et al. 2024; Zhao et al. 2022).

Notably, transport genetics has laid a crucial foundation for resolving this paradox by identifying core regulatory factors through forward genetic screening and CRISPR-mediated editing. However, a critical gap persists: traditional genetics primarily yields static correlations between genotypes and organismal phenotypes. It fails to capture the real-time, environment-dependent choreography of these genetic factors in action. This 'dynamic disconnect' necessitates the integration of transport genetics with modern single-molecule imaging and AI-driven analysis. By leveraging AI-augmented technologies, we can now translate qualitative genetic observations into predictive mathematical models, effectively transforming the 'parts list' of vesicular machinery into a live-action performance of cellular logistics.

This review posits that the era of static observation is giving way to a new paradigm of quantitative prediction, driven by the emergence of multimodal imaging-labeling strategies. We argue that unlocking the black box of vesicular trafficking requires more than higher resolution; an even greater imperative lies in the integration of transport genetics, advanced chemical biology, and computational quantitative analysis. By synthesizing cutting-edge labeling technologies, such as pH-sensitive probes (e.g., pHluorin) for resolving maturation kinetics (Afuwape and Kavalali 2016; Shen and Zhao 2023) and covalent tags (e.g., HaloTag) for high signal-to-noise tracking (Ye et al. 2023), we can now visualize molecular events with unprecedented fidelity.

Crucially, we move beyond visualization to explore quantitative dynamic analysis. AI-augmented imaging technologies allow the translation of genetic observations into predictive models. We discuss how integrating next-generation variants of FRAP and FCS (e.g., DeepFRAP, FCSNet) (Skärström et al. 2021; Tang et al. 2024) with deep learning-enhanced kymograph tools allows for the rigorous mathematical modeling of single-vesicle motility. In particular, alongside breakthroughs in AI algorithms, high-throughput quantification of vesicle velocity, fusion frequency, and colocalization with genetic markers can be progressively realized. This overcomes the limitations of manual analysis and enables the extraction of kinetic parameters directly linked to genetic perturbations. This review aims to construct a framework that maps these nanoscale trafficking events to organismal phenotypes. By providing actionable methodologies to dissect the complex membrane dynamic networks, we offer a roadmap for deciphering plant responses to biotic stress and nutrient cues, ultimately establishing new targets for manipulating vesicular networks in crop improvement.

## From static inventory to dynamic uncertainties: current blind spots in plant trafficking

### The “life-time” paradox of endocytic machinery

While genetic screens have successfully compiled a "parts list" of the plant endocytic machinery, from the evolutionarily ancient AP-2 complex to the plant-specific TPLATE complex (TPC), our understanding of how these components operate in the fourth dimension (time) remains surprisingly fragmented. Current static models depict TPC and AP-2 as stable adaptors that recruit clathrin (Chen et al. 2013; Yan et al. 2021; Zhang et al. 2024). However, this structural view obscures a critical kinetic question: What is the in vivo stoichiometry and dwell time of these complexes at the plasma membrane? Does the octameric TPC assemble incrementally or arrive as a pre-formed unit? And critically, during the fleeting moments of vesicle scission, a process lasting only seconds, how do these complexes dynamically uncoat to allow fusion with the TGN/EE? Traditional confocal microscopy lacks the temporal resolution to capture these sub-second events, leaving a "blind spot" in understanding how plants modulate endocytic rates in response to acute stress, such as the differential regulation of aquaporins under salt shock (Gómez-Méndez et al. 2023). We know who the players are, but we do not yet know the tempo of their performance.

### The “invisible” logic of membrane nanodomains

A similar opacity plagues our understanding of Clathrin-Independent Endocytosis (CIE). We recognize that sterol-rich nanodomains (lipid rafts) act as signaling hubs for immune receptors like FLS2 and FERONIA (Csicsely et al. 2025; Yu et al. 2017). Yet, because these domains (often < 100 nm) fall well below the diffraction limit of conventional optics, their existence has often been inferred rather than directly observed. The central controversy lies in their stability: Are these nanodomains static "islands" awaiting cargo, or are they transient, stimulus-induced clusters that form and dissolve within milliseconds? For instance, while super-resolution snapshots suggest that the RALF1-FER-CAR module reorganizes these domains to exclude immune suppressors (Chen et al. 2023), we lack the continuous, high-speed tracking data to prove this "exclusion model" in real-time. Without determining the diffusion coefficients and confinement radii of these proteins, the "signal specificity" mechanism of plant immunity remains a hypothesis based on static correlations rather than dynamic proof.

### The sorting identity crisis in unconventional secretion

Perhaps the greatest "black box" lies in the secretory pathways, particularly Unconventional Protein Secretion (UPS). Plants have evolved multiple routes to bypass the Golgi, such as EXPO (Wang et al. 2010), MVB-mediated secretion (Liu et al. 2025a), and GDSV pathways (Weng et al. 2025), to deliver leaderless proteins for defense and cell wall remodeling. However, a fundamental sorting logic remains elusive: How does a cytoplasmic protein "know" to enter an EXPO versus an MVB without a canonical signal peptide? Furthermore, in the dense cytoplasmic environment, distinguishing a double-membrane EXPO from an autophagosome or an MVB based solely on morphology is notoriously error-prone. Autophagy-related marker proteins (e.g., ATG8) can assist in distinguishing EXPOs from autophagosomes, providing molecular identifiers for the sorting of UPS pathways (Qi et al. 2023; Zhou et al. 2025a), but reliance solely on these is far from sufficient. Current imaging often relies on long-term overexpression, which may induce artificial aggregation, making it impossible to distinguish physiological secretion from "traffic jams." The field urgently needs a way to map the "trajectory history" of these vesicles, tracking them from biogenesis to fusion, to resolve how multiple UPS pathways coordinate spatially to maintain cell wall integrity under stress.

## Overcoming the optical barrier: advanced labeling strategies for plant cell dynamics

To move beyond the largely static perspectives offered by classical genetics and histology, a new generation of labeling technologies has enabled researchers to visualize vesicular trafficking with unprecedented spatial and temporal resolution. Yet, translating these approaches into plant systems presents significant challenges: thick and optically complex cell walls, high chloroplast autofluorescence, and acidic apoplastic and vacuolar environments. This section evaluates how emerging labeling strategies overcome these plant-specific optical and biochemical constraints, transforming our understanding from a qualitative inventory of components to a quantitative, dynamic view of cellular logistics (Table 1).

**Table 1:** Successful application of different labeling strategies in plants

Method	Marker/Vector Type	Species/Tissue/Background	Representative Plant Examples	References
Isotope Labeling Technique	1) U-13C-pyruvate; 2) ^15^N-labeled compounds	1) *Brassica napus* leaves; 2) *Arabidopsis thaliana*	1) Detection of characteristic isotopomer formation during the "secondary turn" of the TCA cycle in Brassica napus leaves, verifying sustained forward flux under both light and dark conditions; 2) High-precision protein quantification in Arabidopsis	Dellero et al. 2023; Shrestha et al. 2022
pHluorin	1) PEpHluorin 2) PRpHluorin	1) *Arabidopsis thaliana* roots	1) pHluorin-tagged transporters in Arabidopsis thaliana roots enable real-time distinction between plasma membrane-localized populations and internalized populations in acidic endosomes, quantifying the acidification time from TGN to MVB	Shen and Zhao 2023; Martinière et al. 2018
HaloTag	1) Split-HaloTag 2) dye-conjugated ligands	1) *Nicotiana benthamiana*; 2) *Populus*; 3) *Arabidopsis thaliana* (stable transgenic lines)	1) Single-particle tracking of membrane protein nanodomain dynamics in *Arabidopsis thaliana*; 2) Validation of ligand permeability in *Nicotiana tabacum* and *Populus*, enabling long-term dynamic monitoring	Qian et al. 2025; Lang et al. 2006
Fluorescent Timers	1) Dendra2 2) Highlighter	1) *Nicotiana benthamiana* leaves	1) Highlighter system synchronizing cargo transport waves in *Nicotiana benthamiana*, achieving high spatiotemporal precision tracking	Larsen et al. 2023
CRISPR-mediated Bioimaging	1) XVE-Brainbow	1) *Arabidopsis thaliana* regenerated tissues/roots	1) XVE-Brainbow integrated with CRISPR, mapping monoclonal origins of regenerated tissues in *Arabidopsis thaliana*	Lu et al. 2025
Bio-orthogonal Reaction	1) Azide-modified monosaccharides; 2) CarboTag; 3) CuAAC probes	1) *Arabidopsis thaliana* cells/roots	1) Azide-modified monosaccharide labeling of pectin in Arabidopsis thaliana, real-time imaging of dynamic deposition of cell wall components during growth and development; 2) CarboTag combined with click chemistry, quantitative assessment of functional properties of live Arabidopsis thaliana cell walls	Anderson et al. 2012; Besten et al. 2025; Chen et al. 2024a

### Isotope labeling technique: tracing the metabolic flux

Isotope labeling, which uses tracer atoms with identical chemical properties but distinct masses, has long been central to metabolic flux analysis (Derdau and Hesk 2020; Walde et al. 2025). For example, U-13C-pyruvate tracing in Brassica napus leaves revealed the formation of characteristic isotopomers generated during the “secondary turn” of the tricarboxylic acid (TCA) cycle, confirming sustained forward flux under both light and dark conditions (Dellero et al. 2023). Increasingly, isotopic labeling is being integrated into studies of both the conventional (CPS) and unconventional protein secretion pathways (UPS). In plants, ^15^N labeling enables highly accurate protein quantification (Shrestha et al. 2022), while in animal systems, rapid kinetic labeling coupled with LC–MS/MS has been applied to track the secretion of classical cargos such as MMP1 in cancer and stromal cells (Hammond et al. 2018). Drawing on these advances, we propose that combining ^15^N labeling with pharmacological Golgi disruption (e.g. brefeldin A) could generate a proteome-wide map of UPS-dependent cargo release during stress. Such an approach may provide fundamental insights into how pathogen attack reshapes the plant secretome to activate immunity. Although isotope labeling enables the precise tracking of metabolic flux, its inherently low temporal resolution makes it difficult to capture transient UPS events that occur on a second timescale in plants. In addition, the intricate compartmentalization within plant tissues may induce isotope dilution, which impairs the sensitivity of detection. This challenge has stimulated hybrid strategies that couple isotopic precision with rapid fluorescent readouts (e.g. pHluorin or HaloTag reporters), forming multimodal platforms capable of capturing both quantitative fluxes and real-time vesicle behavior. Such integration effectively bridges the gap between molecule-level accuracy and subcellular dynamics, enabling deeper mechanistic insights into UPS function, extracellular vesicle (EV) secretion, and stress-induced cell wall remodeling.

### Fluorescent labeling technique

Fluorescent labeling technologies rely on fluorophores that emit characteristic spectra upon excitation, enabling sensitive and specific detection of target molecules tagged through covalent coupling to recognition elements such as antibodies, enzymes, nucleic acids, peptides, or aptamers (Casto-Boggess et al. 2022; Jun et al. 2022; Maity 2020). These methods allow high-resolution analyses of protein localization (Hao et al. 2024), trafficking (Oppenheimer et al. 2024), interactions (Slavoff et al. 2011), and conformational changes through fluorescent protein tags (Strunk et al. 2009). Below we highlight labeling strategies particularly transformative for plant vesicular trafficking.

#### pHluorin: decoding vesicle maturation via pH gradients

**Fig. 1:**
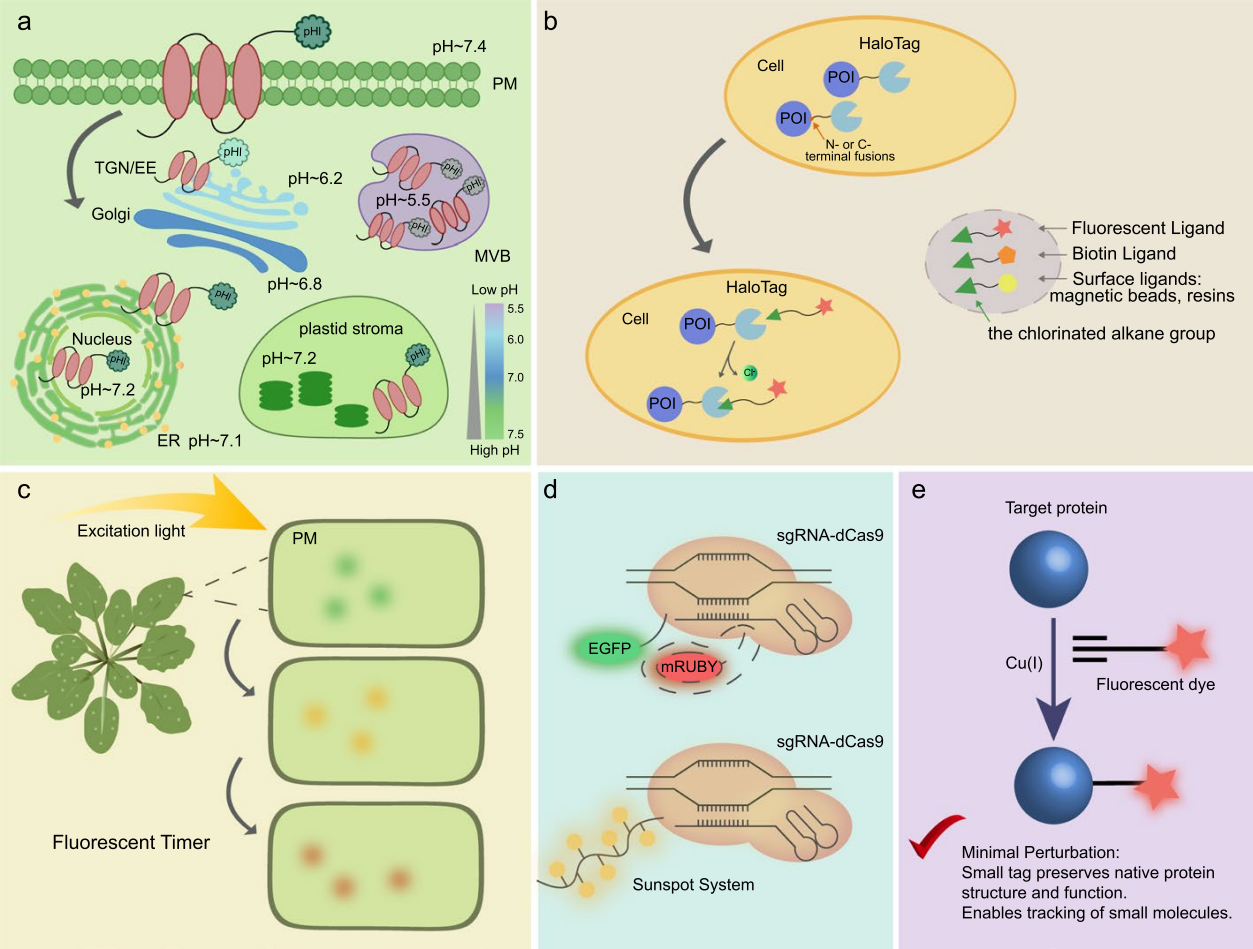
Common fluorescence labeling techniques. **a** pHluorin gradually loses fluorescence as the pH decreases, and exhibits different fluorescence intensities in different organelle compartments due to pH differences. The color scale on the right indicates a continuous gradient from pH 7.5 (green) to pH 5.5 (purple). **b** HaloTag protein binds to different ligands or biotin, enabling imaging of living or fixed cells. **c** As the protein matures, the fluorescent color of the fluorescent timer changes. For example, DsRED-E5 transitions from green through yellow to red, allowing dynamic tracking of protein transport pathways. **d** The CRISPR imaging system: the Cas9 variant dCas9 fused with fluorescent proteins can generate dCas9-EGFP/mRuby, while the CRISPR-Sunspot imaging system established based on the SunTag system is capable of recruiting more fluorescent proteins. **e** Copper-catalyzed azide-alkyne cycloaddition (CuAAC) reaction, conferring the target protein with a small-molecule tag

The lumen of plant endomembrane organelles exhibits a steep pH gradient, ranging from the near-neutral ER (pH ~ 7.1) to the acidic Trans-Golgi Network (TGN, pH ~ 6.1) and the lytic vacuole (pH < 5.5). pHluorin, a pH-sensitive GFP variant, leverages this gradient to act as a "dynamic locator" (Afuwape and Kavalali 2016) (Fig. 1a). pHluorin responsiveness has been further enhanced through engineered modifications, including PEpHluorin (acid-dependent fluorescence intensity modulation) and PRpHluorin (optimized for plant pH quantification), significantly expanding its experimental versatility (Shen and Zhao 2023). In Arabidopsis roots, pHluorin-tagged transporters allow researchers to distinguish between plasma membrane-localized populations and those internalized into acidic endosomes in real-time (Martinière et al. 2018). Beyond simple tracking, advanced derivatives like PEpHluorin enable the precise quantification of vesicle maturation kinetics, measuring the exact time required for a vesicle to acidify during its transition from the TGN to the Multivesicular Body (MVB).Crucially, this technique can distinguish futile cycles from functional trafficking and has been validated in studies across animal systems, such as monitoring the biphasic regulation of synaptotagmin-1 during endocytosis (Chen et al. 2022; Georgiev and Rizzoli 2025). Although the acidic microenvironment of plant cell walls and the extreme acidity of vacuoles (pH < 5.5) induce the quenching of fluorescent signals from conventional pHluorin, next-generation FRET-based ratiometric probes are now overcoming this limitation to map the full endocytic lifecycle (Xu et al. 2025). The effective detection range of pHluorin typically ranges from pH 5.4 to 8.4, and with variants such as PRpHluorin, precise minute scale measurement of vesicular acidification kinetics during the transition from TGN to MVB can be achieved, which serves as a benchmark for quantitative analysis.

#### Halotag: breaking the diffraction and background limit

Self-labeling protein tags (SLPs), including HaloTag, SNAPTag, TMPTag, and CLIPTag, enable covalent protein labeling through dye-conjugated ligands. As a 33 kDa engineered variant of bacterial haloalkane dehalogenase, HaloTag exhibits superior performance characteristics: high fluorescence intensity, minimal background signal, and excellent stability in live-cell applications (Ye et al. 2023) (Fig. 1b). This versatile tool facilitates genetic fusion to either N- or C-termini of target proteins, supporting diverse applications from multicolor imaging and ligand binding assays to endocytosis/exocytosis studies.

The ester bond linkage of HaloTag avoids thioether-mediated photobleaching, thereby endowing it with an inherent high photon budget advantage. Additionally, the elimination of oxidation-prone methionine residues adjacent to the fluorophore via protein engineering, such as the M175L mutation, can further improve the photostability of red and far-red fluorophores severalfold (Ling et al. 2025b). This superior anti-bleaching performance allows HaloTag to withstand the high-intensity excitation light required for imaging deep plant tissues, making it an ideal option for long-term dynamic monitoring in plant tissues. Its plant applications have evolved from initial demonstrations of ligand permeability in tobacco and poplar (Lang et al. 2006) to Split-HaloTag implementations for cytoskeletal protein visualization (Minner-Meinen et al. 2021) and stable transgenic plant applications. HaloTag achieves nanometer-scale resolution in single-particle tracking of membrane protein dynamics in plant systems (Qian et al. 2025) and has proven valuable for CPS/UPS pathway studies through cargo molecule tracking (e.g., PCSK9) and inhibitor validation in animal systems (Lin et al. 2023). Thus, Halotag is not merely a fluorescent protein tag, but also a powerful tool for single-molecule tracking of the behavior of membrane protein nanodomains. Current limitations include the cost of ligands, photodamage potentially induced by excitation light, impaired ligand permeability by plant cell walls, and size-dependent interference associated with organelle targeting. Future development should focus on cost-effective ligands, plant-specific optimization, and photoactivatable systems like PA-JF549 for precise vesicular transport studies.

#### Fluorescent timer: the fourth dimension of trafficking

Static imaging fails to capture protein "age", a vital parameter for distinguishing newly synthesized cargo from recycled pools. Fluorescent Timers (FTs) resolve this by acting as molecular chronometers that shift color during maturation (Fig. 1c). This capability has been pivotal in mapping the sorting fate of Synaptotagmin 1 (Leskova et al. 2019). More recently, the "Highlighter" system, a synthetic optogenetic tool derived from cyanobacteria, has enabled the synchronization of cargo waves with unprecedented spatiotemporal precision (Larsen et al. 2023). However, technical limitations persist. Tandem Fluorescent Timer (TFT) systems suffer from reduced dynamic range and cannot resolve degradation events with half-lives shorter than 30 min (Zhang et al. 2019). Furthermore, environmental fluctuations impair performance; for instance, Dendra2 loses its activity in acidic organelles such as vacuoles (pH < 5.5), rendering it unable to track the process of vesicular degradation. While mCherry maturation in TFTs is highly temperature-dependent, diurnal temperature fluctuations in plants can lead to variations in the maturation time of mCherry. To address these challenges, variants like mEosEM have been developed. This osmium-resistant probe matures within 15 min and withstands chemical fixation, proving ideal for capturing rapid secretory dynamics via SR-CLEM (Fu et al. 2020). Future improvements in stability will enable FTs to transcend simple tracing, serving instead as core metrics to mathematically reconstruct the complete "life history" of membrane proteins under stress.

#### CRISPR-mediated bioimaging: visualizing endogenous reality

Traditional imaging often relies on ectopic overexpression, which can distort protein stoichiometry and induce artificial aggregation. CRISPR-mediated imaging fundamentally shifts this paradigm by visualizing genomic loci and transcripts at their endogenous levels (Fig. 1d). By fusing catalytically inactive dCas9 with fluorescent markers, this approach enables the non-intrusive tracking of chromosomal dynamics in real time (Khosravi et al. 2020). A critical breakthrough in sensitivity is the CRISPR-SunTag system, which recruits multiple fluorophores to a single target. This signal amplification allows for the single-molecule detection of low-abundance mRNAs that were previously invisible to standard dCas9-EGFP systems (Sun et al. 2020). Furthermore, integrating CRISPR with lineage tracing tools (e.g., XVE-Brainbow) has successfully mapped the monoclonal origins of regenerated tissues in Arabidopsis (Lu et al. 2025). Of course, certain challenges remain for its application in plant tissues. For instance, the plant genome contains a high abundance of repetitive sequences, which results in a relatively high off-target binding rate of dCas9 and thus elevates background noise. A greater challenge is the chloroplast autofluorescence in green tissues, which severely interferes with the signal detection of endogenous low-abundance mRNAs. However, the adoption of far-red fluorescent probes (e.g., iRFP670) and two-photon excitation technology is rapidly overcoming these optical barriers (Potlapalli et al. 2023). By combining CRISPR with multimodal imaging platforms, researchers continue to expand its utility in decoding the spatiotemporal organization of genomic elements in plant systems.

### Bio-orthogonal reaction:overcoming steric hindrance

While fluorescent proteins are powerful, their substantial size (~ 27 kDa) creates steric hindrance that can disrupt the native behavior of small lipids and glycans. Bio-orthogonal chemistry, also known as "click chemistry", circumvents this by using diminutive, inert tags that react exclusively with specific probes. This precision allows for the in situ visualization of metabolic fluxes and cell wall remodeling without perturbing cellular physiology (Bird et al. 2021; Liu et al. 2022). This approach leverages engineered protein tags that selectively react with complementary probes (Scinto et al. 2022), enabling unprecedented resolution in studying protein localization, conformational dynamics, and interaction networks. In terms of quantitative performance, click chemistry, can meet the localization and quantification requirements for plant cell membrane nanodomains when combined with super-resolution microscopy (e.g., STED), while maintaining a fluorescent signal half-life of at least 4 h after labeling, which satisfies the demand for long-term monitoring of plant trafficking dynamics (Anderson et al. 2012). Crucially, the application of click chemistry in plant science has expanded beyond proteins to the dynamic tracing of cell wall polysaccharides, overcoming the penetration limitations of bulky antibodies. For instance, metabolic labeling enables the incorporation of azide-modified monosaccharide analogs into cell wall polysaccharides, followed by the coupling of fluorescent probes via bio-orthogonal click reactions, which allows real-time visual imaging of the dynamic deposition and remodeling of cell wall components such as pectin during plant growth and development (Chen et al. 2024a). Furthermore, novel modular tools (e.g., Carbo Tag) utilize pyridinylboronic acid moieties to form reversible covalent conjugations with vicinal diol moieties in the cell wall (e.g., those in pectin and alginate). Combined with click chemistry-modified fluorescent probes of distinct emission wavelengths, these tools enable the dynamic quantitative assessment of functional properties (e.g., network porosity and pH) of the cell wall in living plants at the subcellular level (Besten et al. 2025).

The copper-catalyzed azide-alkyne cycloaddition (CuAAC), the foundational click chemistry reaction, has become particularly valuable for biomolecular tracking both in vitro and in vivo due to its remarkable efficiency and substrate versatility (Bishnoi et al. 2025) (Fig. 1e). Although the cytotoxicity of copper catalysts has historically been a limitation, recent innovations in ligand-assisted catalysis have significantly mitigated these side effects (Nian et al. 2025). This technology is now poised to reveal the nanoscale dynamics of the plant cell surface, the critical frontier of pathogen interaction, providing a resolution that genetically encoded probes cannot achieve (Qiu et al. 2021; Wang et al. 2024). This paradigm has redefined in vivo labeling/imaging, providing unprecedented resolution for intracellular analysis. Specifically, this technology effectively addresses the key challenge of visualizing dynamic molecular events (such as metabolic flux) in their native physiological environments without introducing the structural steric hindrance commonly associated with bulky labels. But its application in plants is not without obstacles. Plant cells are more sensitive to Cu^2^⁺, with a concentration exceeding 50 μM significantly inhibiting root growth (Lequeux et al. 2010); cellulose and pectin in plant cell walls can non-specifically bind to azido/alkynyl probes, thus markedly elevating the background signal. Future innovations should optimize bio-orthogonal platforms for real-time monitoring of biomacromolecule endocytosis/exocytosis, potentially through integrated approaches that combine complementary analytical techniques to elucidate complex biological phenomena in cellular/plant systems and drive breakthroughs in basic life sciences and applied plant physiology.

## Quantitative bioimaging analysis: from visualization to prediction

The true revolution in plant cell biology lies in the shift from qualitative observation to quantitative prediction. Advanced computational tools now allow us to extract precise kinetic parameters, such as diffusion coefficients, dwell times, and transport velocities, transforming microscopic images into mathematical models of cellular logic.

### DeepFRAP: unpacking the “black box” of deep learning kinetics

**Fig. 2:**
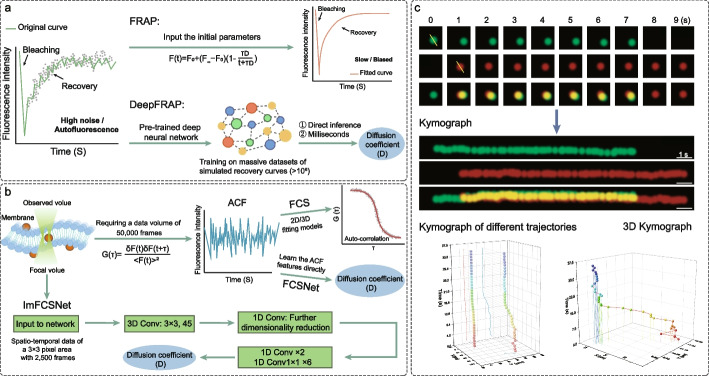
Common molecular dynamics quantification. **a** The result curves of FRAP only undergo simple data correction without complex model optimization, directly reflecting the original dynamic process of fluorescence recovery in experiments. They are susceptible to experimental errors, and the curve morphology may exhibit fluctuations and irregularities. In contrast, the result curves of DeepFRAP are optimized curves processed by deep learning models, featuring a smoother morphology and higher accuracy. **b** FCS is a method that analyzes the original image and obtains the autocorrelation function (ACF) curve based on the G(τ) formula (where τ is the delay time and F(t) is the fluorescence intensity of a certain pixel at time t), and fitting the curve requires a considerable amount of time. FCSNet directly predicts the diffusion coefficient (D) from the ACF curve without manual fitting. ImFCSNet does not rely on the ACF curve. It first compresses the 3 × 3 pixel spatiotemporal data into 45 temporal feature maps through a 3D convolutional layer, then further reduces the dimensionality with a 1D convolutional layer. After extracting deep features through two groups of 1D convolutional residual blocks and six groups of Conv1 × 1 1D residual blocks, it performs global average pooling along the time dimension on the feature maps and then connects to a fully connected layer to output the result. ImFCSNet requires less data when outputting the diffusion coefficient and has a faster analysis speed. **c** The Kymograph continuously stacks images from each time point along the time axis, enabling intuitive visualization of the spatiotemporal distribution dynamics of different components. Meanwhile, the output curves can capture the changes in fluorescence intensity of these components throughout the entire time course, and quantitatively characterize the temporal fluctuation patterns of their signals

Fluorescence Recovery After Photobleaching (FRAP) has long served as the gold standard for measuring protein mobility and molecular dynamics in plant cell biology (Fig. 2a). It has been extensively applied to dissect diverse physiological processes (Deng and Wan 2024; Liu et al. 2024; Wang et al. 2025a), ranging from the trafficking of cellulose synthase complexes (CESA6) at the plasma membrane (He et al. 2018) and the dynamic condensation of Rubisco in chloroplasts (Chen et al. 2025a), to the liquid–liquid phase separation of secretory granules (SgII) in neuroendocrine cells (Lin et al. 2022) and actin turnover under mechanical stress (Skamrahl et al. 2019). However, the traditional analysis of these FRAP datasets relies heavily on Non-linear Least Squares (NLS) fitting (Skärström et al. 2021).This mathematical approach forces experimental data to conform to idealized analytical equations (e.g., the Soumpasis or Axelrod models) and faces two critical bottlenecks in plant research: first, the optical heterogeneity of plant tissues, characterized by refractive cell walls and large vacuoles, introduces complex noise that often traps NLS algorithms in local minima, leading to erroneous diffusion coefficient (D) estimates; second, the dependency on manual initial parameter guesses introduces operator bias and precludes the automation required for large-scale genetic screening.

Recent advances, such as DeepFRAP (Skärström et al. 2021), represent a paradigm shift from traditional iterative fitting to data-driven inference. By training on massive datasets of simulated recovery curves (> 10^6^), these AI models may be able to "see through" the high noise characteristic of plant tissues (e.g., cell wall scattering), extracting relatively accurate kinetic parameters in milliseconds while reducing the operator bias often associated with manual initial parameter guesses (Fig. 2a). Crucially, these training datasets are synthetically corrupted with varying levels of Poisson and Gaussian noise to mimic realistic experimental conditions (Skärström et al. 2021).Through this training, the neural network learns to recognize the underlying kinetic "features" of diffusion hidden within noisy data, effectively establishing a high-dimensional mathematical mapping function f(x) = y, where input x is the raw, noisy recovery curve and output y is the precise kinetic parameter.

This architecture offers distinct advantages for plant research by fundamentally changing how data is processed (Table 2). In terms of quantitative metrics, unlike NLS, which treats noise as an error to be minimized, DeepFRAP provides noise-robust feature extraction, trained to "see through" fluctuations caused by low signal-to-noise ratios (SNR). This capability allows for accurate estimates even when recovery curves are distorted by chloroplast autofluorescence or cell wall scattering. Furthermore, by eliminating iterative convergence loops, the method achieves instantaneous high-throughput screening, reducing computation time by orders of magnitude (milliseconds vs. seconds per cell). This speed enables quantitative phenotyping on a population scale, allowing researchers to screen hundreds of mutant lines to pinpoint subtle vesicular transport defects that would be statistically invisible to manual analysis (Skärström et al. 2021).

**Table 2:** Summary of deep learning quantitative analysis tools for plant bioimaging

Tool Name	Input Requirements	Output Parameters	Time Efficiency	Open Source
Deep Learning-enhanced FRAP (DeepFRAP)	1) Raw FRAP recovery curve with plant tissue noise 2) Laser bleached region parameters	Direct 1) Diffusion coefficient (D) 2) Initial Concentration (c_0_) 3) Bleaching parameter (α) Indirect 1) Noise Variance (a) 2) Reconstructed recovery curve	1) Single-cell analysis is accomplished at the millisecond scale 2) It features high-throughput screening capability	Yes; All data and code have been made publicly available (Skärström et al. 2021)
FCSNet	1) Autocorrelation Function (ACF) curve of Fluorescence Correlation Spectroscopy (FCS)	1) Diffusion coefficient (D)	1) Instantaneous prediction 2) No need for the selection and fitting of complex mathematical models	Yes; The training code for the model is hosted on public GitHub repositories (Tang et al. 2024)
ImFCSNet	1) Raw fluorescence intensity trajectories (3 × 3 pixel spatiotemporal data) 2) Excitation light parameters	1) Diffusion coefficient (D)	1) Data demand is reduced by a factor of 20 (2,500 vs 50,000 frames) 2) Enable the construction of real-time kinetic profiling	Yes; The training code for the model is hosted on public GitHub repositories (Tang et al. 2024)

### FCS and FCSNet: single-molecule sensitivity

To probe molecular dynamics below the diffraction limit, Fluorescence Correlation Spectroscopy (FCS) analyzes the spontaneous intensity fluctuations arising from the transit of single fluorophores through a microscopic detection volume (Fig. 2b). Unlike FRAP, which measures the ensemble relaxation of a macroscopic population, FCS provides single-molecule sensitivity, enabling the precise quantification of diffusion coefficients, molecular concentrations, and oligomerization states (Cui et al. 2018).This capability has been instrumental in dissecting complex physiological processes in plants, such as determining the assembly stoichiometry of membrane proteins like ammonium transporters (AMT1;3) (Wang et al. 2018) and receptor kinases (BRI1) (Wang et al. 2015). Furthermore, FCS has proven indispensable for characterizing the internal fluidity of biomolecular condensates, such as verifying the liquid-like behavior of secretogranin II (SgII) droplets through the analysis of molecular exchange rates in animal studies (Lin et al. 2022). In fact, the application of FCS in plant tissues is inevitably constrained: plant cell wall scattering gives rise to an increased estimation error of the diffusion coefficient, and the cytoplasmic viscosity of plant cells is higher than that of animal cells, which renders it difficult to accurately discriminate molecules with low diffusion rates. More crucially, traditionally, obtaining quantitative kinetic maps in plants required prohibitively long acquisition times (> 50,000 frames), leading to phototoxicity. However, recent deep learning innovations like ImFCSNet (Sankaran and Wohland 2023; Tang et al. 2024) have broken this barrier by extracting diffusion coefficients directly from raw intensity traces using as few as 2,500 frames (Table 2). This effectively reduces light exposure by 20-fold, making live-cell quantitative mapping feasible even in light-sensitive plant tissues (Fig. 2b). Moreover, ImFCSNet exhibits remarkable resilience to optical aberrations, maintaining accurate parameter estimation even in moderately defocused samples, which is a frequent challenge when imaging deep within refractive plant tissues or thick organs like Drosophila embryos (Tang et al. 2024). By democratizing data evaluation and enabling real-time parameter mapping without the need for complex analytical models, deep learning FCS provides a robust framework for quantifying the spatiotemporal heterogeneity of membrane proteins and phase-separated condensates in living plant cells (Sankaran and Wohland 2023).

To address specific biological questions regarding nanoscale organization that standard confocal FCS cannot resolve, these computational advances are increasingly paired with super-resolution modalities. For example, Stimulated Emission Depletion-FCS (STED-FCS) shrinks the observation volume below the diffraction limit (< 50 nm), allowing researchers to distinguish between free diffusion and transient trapping within nanodomains, as demonstrated with lipid analogs and GPI-anchored proteins (Sezgin et al. 2019). Similarly, Fluorescence Cross-Correlation Spectroscopy (FCCS) extends this functionality to two channels, enabling the simultaneous detection of co-diffusion and protein–protein interactions (Yu et al. 2020). The future of quantitative plant cell biology lies in the synergistic integration of these high-end optical techniques with deep learning data processing, establishing a workflow where "unseen" molecular dynamics are translated into predictive mathematical models of cellular function. To demonstrate this via a worked conceptual example, we propose an ImFCSNet-based framework where the measured diffusion coefficient (D) of nutrient transporters serves as a proxy for their functional assembly state. In this scenario, a transition from high-velocity Brownian motion to a reduced, confined diffusion state indicates the active recruitment of transporters into membrane nanodomains, which constitutes the rate-limiting step for nutrient assimilation. For instance, in the cases of ammonium transporters (e.g., AMT1;3) or receptor-like kinases (e.g., BRI1), a quantified reduction in the measured diffusion coefficient (D) indicates an increased degree of oligomerization or ligand-induced clustering. By incorporating these real-time kinetic shifts into a predictive physiological model, researchers can quantitatively link the fraction of "trapped" receptors to measurable macroscopic outputs, such as the specific nitrogen uptake rate or the cumulative root elongation speed, thereby effectively bridging the gap between nanoscale stochastic motion and deterministic organ-level development.

### Kymograph analysis: spatiotemporal fingerprinting

Kymograph analysis represents a critical reductionist approach in quantitative bioimaging, compressing complex multidimensional video data into intuitively interpretable 2D space–time plots (Fig. 2c). By converting time-lapse image sequences into topological representations-where spatial axes capture positional changes and the temporal axis documents dynamic evolution-kymography provides a visual and mathematical "fingerprint" of molecular motility (Jakobs et al. 2019).This technique is uniquely powerful for extracting kinetic parameters that are often obscured in bulk measurements, such as molecular residence time, linear velocity, and transient colocalization events. Recently, the development of Single-Molecule (SM) kymography based on Variable Angle TIRFM (VA-TIRFM) has further refined this capability, allowing for the transformation of 2D particle trajectories into 3D (2D + t) volumes. This allows researchers to rotate the data visualization to identify optimal projection angles, thereby avoiding the misestimation of dwell times caused by irregular particle trajectories on the curved plasma membrane (Su et al. 2023).

In the context of plant signal transduction, kymography has evolved into a definitive tool for distinguishing between competing mechanistic models. For instance, in the study of the G-protein regulator AtRGS1, kymographic analysis revealed that different immune signals trigger distinct spatiotemporal "signatures." While the PAMP flg22 induces rapid, short-lived endocytic events associated with the Clathrin-Mediated Endocytosis (CME) pathway, the DAMP Pep1 triggers different trajectory patterns linked to Sterol-Dependent Endocytosis (SDE). These distinct kymographic footprints provided the first direct evidence that plants utilize specific endocytic routes to discriminate between microbial and endogenous danger signals (Su et al. 2024). Similarly, in phototropism research, kymography was instrumental in linking protein mobility to photoreceptor activation; it revealed that blue light irradiation induces a dose-dependent increase in the lateral diffusion and dimerization of phototropin 1 (phot1) at the plasma membrane, a dynamic shift that is notably absent in kinase-inactive mutants (Xue et al. 2018).

Beyond single-molecule tracking, kymography is increasingly applied to decipher macroscopic physiological rhythms and biophysical phase transitions. At the tissue level, kymographs have successfully mapped the oscillatory expression of NSP1 and cytokinin response reporters in *Lotus japonicus* roots, revealing a periodic "root clock" with 6-h intervals that regulates rhizobial infection zones (Soyano et al. 2024). At the biophysical level, kymographic analysis of DNA condensates has identified a novel "ballistic wave" diffusion mechanism, characterized by a linear relationship between front displacement and time (ΔX ∝ t), which fundamentally differs from the classic Fickian diffusion (ΔX ∝ t1/2) observed in standard liquid–liquid phase separation (Chen et al. 2025b). It is undeniable, however, that the unique characteristics of plant tissues also pose challenges to the application of kymography. In plant cells, particularly in pollen tubes, the vesicle density exceeds 100 per μm^2^, which leads to an elevated trajectory crossing rate and thus greatly increases the error in manual analysis. Quantitatively, manual tracing of kymographs for such high-density vesicle populations requires several hours of work per cell, with the error rate being substantially elevated by trajectory crossing. In mesophyll cells, chlorophyll autofluorescence reduces the signal-to-background ratio, making it difficult to identify weakly fluorescent vesicles. Furthermore, despite its versatility, traditional kymography remains labor-intensive, often requiring manual verification to distinguish signal from noise in complex backgrounds. Future developments will likely focus on integrating automated, deep learning trace linking to resolve crossing trajectories and improve throughput for high-density particle tracking (Su et al. 2023).

### Hybrid modality integration: the ultimate resolution

To simultaneously achieve high spatiotemporal resolution localization and characterization of dynamic behaviors of biomolecules or subcellular structures, thereby facilitating comprehensive mechanistic investigations, hybrid strategies have emerged as a progressive solution by synergizing the strengths of complementary techniques. In addition to the common integration of electron microscopy and optical imaging techniques for resolving the spatial distribution of vesicles (Liu et al. 2025b), integrated approaches incorporating kinetic analysis have also been increasingly adopted. A prime example of this integration is the development of Line Interleaved Excitation Scanning STED-FCS (LIESS-FCS), which overcomes the limitations of traditional spot-variation analysis that requires sequential measurements prone to biological drift. By quasi-simultaneously acquiring confocal and super-resolution STED-FCS data in a single measurement, LIESS-FCS allows for the instantaneous determination of diffusion laws (Schneider et al. 2018). Both STED-FCS and LIESS-FCS enable the quantitative discrimination of transient entrapment, regional partitioning and free diffusion of receptors and adaptor proteins in intracellular membrane nanodomains by analyzing the deviation of the diffusion time across varying observation areas. Specifically, the heterogeneity of diffusion coefficients and the distinct intercepts of diffusion laws allow researchers to differentiation between the unrestricted free diffusion of receptors and their sub-diffusive transient entrapment within nanodomains upon binding to adaptor proteins. Furthermore, spatial heatmaps generated from these modalities can be employed to realize regional partitioning, while the integration of dual-channel cross-correlation permits the quantification of the regional colocalization efficiency and interaction stability between receptors and their adaptors. This capability is critical for dissecting the heterogeneity of plant plasma membranes, as it can rigorously distinguish between nanoscopic "trapping" mechanisms, such as those regulating sphingolipid dynamics, and "domain" incorporation characteristic of GPI-anchored proteins (GPI-APs) (Schneider et al. 2018).

Moving from single-point precision to expansive spatial quantification, a further breakthrough was achieved with the introduction of Oblique Line Scanning (OLS) microscopy. OLS illumination generates a light-sheet-like excitation volume that drastically improves the signal-to-noise ratio (SNR) while minimizing phototoxicity, addressing the long-standing challenge of quantifying weakly labeled targets in living cells (Driouchi and Johnson 2025). Critically, this modality serves as a versatile platform for hybrid quantitative analysis; by enabling high-speed OLS-FCS and OLS-FRAP, researchers can now measure single-protein interaction kinetics and oligomerization states across large cellular fields with subcellular precision, rather than being restricted to isolated points (Driouchi and Johnson 2025).These multimodal strategies fundamentally redefine our analytical capacity, enabling the dissection of complex processes, from nanoscale membrane organization to macroscopic vesicular trafficking, with unprecedented molecular specificity and spatiotemporal fidelity. However, the limitations they may encounter in plant tissues should also be taken into account. In thicker plant tissues (e.g., stems), the signal-to-noise ratio (SNR) of OLS microscopy can be significantly reduced. Furthermore, it is also a critical issue whether the combination of multiple techniques will cause the accumulation of phototoxicity, leading to a marked decline in cellular viability. Thus, achieving the "ultimate resolution" in the study of plant vesicle trafficking is not merely accomplished by simply stacking advanced techniques; instead, it requires tailoring the integration strategy based on the unique physiological and optical characteristics of plant systems.

## Conclusions and perspectives

The exploration of plant vesicular trafficking is currently undergoing a fundamental paradigm shift, transitioning from the qualitative description of static components to the quantitative prediction of dynamic networks (Driouchi and Johnson 2025). As highlighted in this review, the synergistic integration of multimodal imaging with deep learning quantitative analysis is the key to unlocking the long-standing "black box" of membrane logistics. In the field of traditional quantitative cell biology, we commonly adopt approaches such as Bimolecular Fluorescence Complementation (BiFC), Fluorescence Resonance Energy Transfer/Fluorescence Lifetime Imaging (FRET/FLIM), and TurboID-based proximity labeling to detect protein–protein interactions within the plant cell's endomembrane system in real time (Feng et al. 2024; Zhou et al. 2023). More recently, Split-YFP-coupled Split-TurboID has emerged as a robust modality not only for detecting protein interactions but also for the quantitative analysis of the interacting proteome spatially confined to specific endomembrane subdomains (Huang et al. 2025). These proximity-based quantitative methods provide essential chemical and molecular dimensions that complement multimodal imaging and quantitative tracking technologies; while AI-driven kinetics resolve the "tempo" of molecular motility, proximity labeling defines the "spatial neighbors" and interaction networks. Such integration lays the foundation for researchers to construct comprehensive atlases of nanoscale molecular dynamics, effectively bridging the gap between stable biochemical complexes and transient membrane behavior. In this review, we specifically focus on the synergistic integration of these frameworks with deep learning-enhanced quantitative tracking. By combining chemical biology tools such as pH-sensitive sensors and bioorthogonal tags with computational modeling, we can now resolve the spatiotemporal characteristics of molecular events previously undetectable by conventional microscopy and acquire precise kinetic parameters including diffusion coefficients, residence times and vesicle binding efficiency (Michelis et al. 2022; Zhang et al. 2020).

These vesicle kinetic parameters exhibit a well-defined correlative relationship with plant stress-resistant phenotypes, thus providing direct guidance for breeding practices. Vesicle kinetic parameters such as diffusion coefficient (D) and molecular residence time can be further converted into quantitative biomarkers, which serve as core indicators for the screening of stress-tolerant cultivars. For instance, under salt stress, the diffusion coefficients of aquaporin vesicles in salt-tolerant genotypic plants are typically maintained within a specific range, a hallmark of their efficient membrane trafficking and recycling capacity. In the case of biotic stress, the residence time of immune receptors in membrane nanodomains is directly correlated with the efficiency of disease-resistant signal transduction; the threshold values of these parameters can therefore act as quantitative criteria for breeding screening. By integrating AI-driven high-throughput imaging with molecular markers linked to these "dynamic traits," breeders can utilize trafficking kinetics as functional pQTL (protein Quantitative Trait Loci) to identify superior alleles. This approach overcomes the tardiness and subjectivity inherent to traditional phenotypic identification, thereby enabling the implementation of precision breeding.

Despite these advances, the unique optical landscape of plant tissues, characterized by refractive cell walls and high autofluorescence, remains a formidable barrier. To fully bridge the gap between microscopic events and macroscopic phenotypes, future research must prioritize three strategic directions:

First, the development of plant-optimized labeling toolkits. This includes engineering brighter, photostable probes (e.g., near-infrared dyes) that can penetrate deep tissues and withstand the acidic environments of the apoplast and vacuole, as well as cost-effective, bio-orthogonal ligands to democratize technologies like HaloTag in crop research.

Second, the deep integration of Artificial Intelligence. We must move beyond simple image enhancement to leverage deep learning architectures (e.g., DeepFRAP, ImFCSNet) that can infer precise kinetic parameters from noisy, low-light data (Maizón and Barrantes 2022). Furthermore, developing interpretable AI models will be crucial for decoding the complex rules governing cargo sorting and vesicle interactions under multifactorial stress conditions (Li et al. 2024; Zhou et al. 2025b). Notably, deep learning models exhibit critical limitations in plant cell research involving rare biological outliers and discrepancies between simulated and experimental data. Overreliance on complete training datasets may leads to "model hallucinations" or forced fitting of low-frequency, non-canonical vesicular trafficking events (e.g., stress-induced transient aggregation and abnormal fusion), resulting in the loss of key biological insights. Meanwhile, pre-training on synthetic data simulated with Gaussian or Poisson noise fails to capture the high heterogeneity of real experimental data, such as tissue-specific cell wall properties, stochastic fluctuations in chloroplast autofluorescence, and cell type-dependent variations in cytoplasmic viscosity. This discrepancy creates a prominent "Domain Gap", where a model trained on simplified simulations may inaccurately estimate kinetic parameters when confronted with the complex optical aberrations and non-Fickian diffusion typical of living plant tissues. Thus, developing robust domain adaptation algorithms and active learning strategies for rare event capture will emerge as the core future technological direction to elucidate the dynamics of plant cells.

Third, multidimensional cross-scale synthesis. The ultimate goal is to map nanoscale membrane dynamics to whole-plant physiology. This requires coupling live-cell imaging with single-cell transcriptomics and proteomics to visualize how vesicular networks reconfigure in specific cell types during pathogen attack or nutrient deprivation. The core of this interconnection resides in the integration of cascade regulation and feedback adaptation along the signal-transcription-vesicle axis: First, immune or nutrient signals drive transcriptional reprogramming in cells; by regulating the expression of vesicle-associated genes such as RAB GTPases or clathrin subunits, they directly define the functional parameters of vesicular trafficking, tailoring its dynamics, efficiency and specificity to the demands of the ongoing cellular response (Jin et al. 2021). At the single-cell level, these transcriptional states dictate the abundance of trafficking machinery, while live-cell imaging parameters-such as the diffusion coefficient (D) or membrane dwell time-reflect the real-time operational efficiency of these gene products in response to stimuli. In turn, vesicles retrogradely modulate the activity of transcriptional networks via the translocation of signaling molecules and cargoes, forming a bidirectional regulatory circuit that ultimately enables precise cellular responses to immune threats or nutrient fluctuations (Zhang et al. 2025). By correlating single-cell RNA-seq "transcriptional clusters" with AI-mapped "trafficking signatures," appropriate integration strategies can elevate the dissection of the underlying mechanisms from static gene expression profiles to dynamic membrane-nuclear feedback circuits, providing a quantitative framework to predict how plants orchestrate organismal-level resilience through localized cellular logistics.

As we advance these powerful technologies, we must also adhere to responsible scientific practices, carefully navigating ethical considerations regarding CRISPR off-target effects and phototoxicity thresholds. Furthermore, as plant imaging research shifts from qualitative description to precise quantitative characterization, cross-laboratory comparability of experimental results has become a critical unresolved challenge. Variations in microscopic imaging hardware and the high sensitivity of AI models (e.g., DeepFRAP for simulated training data) across studies often render kinetic parameters non-comparable. To this end, we call on the plant research community to jointly establish standard benchmark datasets for plant cellular dynamics and a standardized reporting format for imaging metadata. This standardization will not only validate the cross-platform robustness of deep learning models but also lay the foundation for constructing a universally applicable plant cellular dynamics atlas. Ultimately, establishing this quantitative framework will not only rewrite the textbooks of plant cell biology but also provide actionable targets for precision breeding. By regulating the cellular core signal transduction and material transport hub during vesicle transport, we provide the possibility of developing a new generation of crops with stronger stress resistance and more efficient nutrient utilization capabilities, which will help ensure agricultural productivity under changing climatic conditions.

## Data Availability

No datasets were generated or analyzed during the current study.
